# Professional factors supporting workaholism among Tunisian engineers

**DOI:** 10.1192/j.eurpsy.2022.623

**Published:** 2022-09-01

**Authors:** A. Bouaziz, R. Sallemi, M. Bouhamed, R. Masmoudi, I. Feki, J. Masmoudi

**Affiliations:** 1 Hospital university of HEDI CHAKER, Psychiatry A Department, Sfax, Tunisia; 2 Hedi chaker hospital, Psychiatrie, Sfax, Tunisia; 3 Hospital Hédi Chaker, Sfax, Sfax, Tunisia

**Keywords:** determinants, work addiction, engineers, professional factors

## Abstract

**Introduction:**

Workaholism or work addiction is a growing public health that may induce negative consequences on professional life. Engineers are at risk given the globalization and increased competition in their jobs.

**Objectives:**

The aim of the study was to assess the different professional factors that promote wokaholism among Tunisian engineers.

**Methods:**

A cross-sectional descriptive and analytical study conducted among Tunisian engineers during July 2021. The data were collected by an online questionnaire including the socio-demographic and professional information and the “the Work Addiction Risk Test” (WART) which was used to assess the workaholism.

**Results:**

Participants were 52 engineers (31 males and 21 females), and aged from 23 to 55 years old (average age 30.75 years). Thirty-five engineers (67.3%) were single. Concerning professional data, 30.8% of engineers worked in the public and 51.9% of them were computer engineers. Of the participants, 11.7% worked more than 12 hours, 61.5% worked overtime and 92.3% had weekly rest. The prevalence of workaholism in Tunisian engineers was 23.1%. Engineers working in the public sector and working more than 12 hours had significantly higher proportion of work addiction with p <0.001 and p = 0.01, respectively. However, no significant difference was found by specialty, working overtime and having weekly rest according to workaholism.

**Conclusions:**

In our study, we found that the public work sector and extended working hours promote work addiction. Addressing supporting factors in the work environment and periodic examination of the engineers and responding accordingly is required.

**Disclosure:**

No significant relationships.

